# Comparison of Antivirulence Activities of Black Ginseng against Methicillin-Resistant *Staphylococcus aureus* According to the Number of Repeated Steaming and Drying Cycles

**DOI:** 10.3390/antibiotics10060617

**Published:** 2021-05-21

**Authors:** Young-Seob Lee, Kwan-Woo Kim, Dahye Yoon, Geum-Soog Kim, Dong-Yeul Kwon, Ok-Hwa Kang, Dae Young Lee

**Affiliations:** 1 Department of Herbal Crop Research, National Institute of Horticultural and Herbal Science, RDA, Eumseong, Chungbuk 27709, Korea; youngseoblee@korea.kr (Y.-S.L.); swamp1@korea.kr (K.-W.K.); dahyeyoon@korea.kr (D.Y.); kimgs0725@korea.kr (G.-S.K.); 2 Department of Oriental Pharmacy, College of Pharmacy and Wonkwang Oriental Medicines Research Institute, Wonkwang University, Iksan 54538, Korea; sssimi@wku.ac.kr (D.-Y.K.); okey@wku.ac.kr (O.-H.K.)

**Keywords:** *Staphylococcus aureus*, MRSA, black ginseng, *Panax ginseng*

## Abstract

Korean ginseng has been widely used in Eastern medicine for thousands of years. The contents of the compounds in ginseng roots change depending on the amount of steaming and drying, and the drying method used. Black ginseng (BG) is the Korean ginseng processed by repeated steaming and drying. In this study, 5-year-old fresh Korean ginseng roots were steamed and dried 3 or 5 times, and we investigated how many cycles of steaming and drying are preferable for antivirulence activities against methicillin-resistant *Staphylococcus aureus* (MRSA). As a result, the antivirulence activities was increased by the treatment of BG that was steamed and dried three times, and the effect was further increased by five-time processed BG. Moreover, an ELISA showed that the TNF-α production of RAW264.7 cells stimulated by MRSA supernatants was inhibited by subinhibitory concentrations of BG extract. The expression of Hla, staphylococcal enterotoxin A (SEA), and staphylococcal enterotoxin B (SEB), an important virulence factor in the pathogenicity of MRSA, was found to decrease when bacterial cells were treated with BG extract. The antivirulence activities of BG were not simply due to pathogen growth inhibition; the BG extract was shown to decrease *agrA*, *hla*, *sea*, and *seb* expression in MRSA. Therefore, BG strongly reduces the secretion of the virulence factors produced by *Staphylococcus aureus*, suggesting that a BG-based structure may be used for the development of drugs aimed at staphylococcal virulence-related exoproteins. This study suggests that BG could be used as a promising natural compound in the food and pharmaceutical industry.

## 1. Introduction

*Staphylococcus aureus* (*S. aureus*) is a common bacterial genus in hospitals and communities found on the anterior nares of 20 to 80% of the human population [1,2]. Preventing and controlling the spread of the antibiotic resistance of this pathogen requires numerous actions of prevention, and infection control is now essential. The rapid global emergence of resistant pathogenic bacteria that cause fatal infectious diseases such as methicillin-resistant *S. aureus* (MRSA) is occurring, endangering the efficacy of antibiotics used in medical institutions [3,4]. This pathogen secretes toxins and exoenzymes that exacerbate acute infections such as sepsis, skin abscesses, and food poisoning, and toxins are a means of aggression by harmful bacteria that damage human tissue and cells [5,6].

α-Hemolysin (α-toxin, Hla), also known as a-toxin, is a 33 kDa pore-forming protein of a water-soluble monomer that is encoded by a single genetic locus, *hla*. Hla, which oligomerizes to a prepore structure, assembles into a homoheptamer. Then, Hla penetrates the membrane of the target cell by the extrusion of the β barrel and forms a hydrophilic transmembrane channel in the lipid bilayer of the membrane. Pore formation causes leakage of molecules smaller than 2 kDa, such as K^+^ and Ca^2+^ ions, inducing the necrotic death of the target cell [7,8,9]. Staphylococcal enterotoxins (SEs) secreted by *S. aureus* are bacterial exotoxins that cause staphylococcal food poisoning (SFP) [10]. SEs are low-molecular-weight (23–29 kDa) single-chain basic globular proteins [11]. SEs affect intestinal cells, which induces gastroenteritis, typically evoking vomiting, diarrhea, and intestinal or gastric inflammation [10,12]. These are also part of the family of superantigens (SAgs) because of their activity leading to the excessive activation of T cells, with a subsequent enormous release of cytokines, invoking a life-threatening toxic-shock syndrome [10]. The most common staphylococcal enterotoxins are staphylococcal enterotoxin A (SEA) and staphylococcal enterotoxin B (SEB) [12]. The accessory gene regulatory (Agr) system controls virulence factor gene expression by sensing the accumulation of autoinducing peptide via response regulator AgrA and histidine kinase AgrC [13]. The Agr locus has a direct positive impact on Hla expression [14].

Recently, antivirulence studies of medicinal plants used as traditional medicines were attempted as a potential source of drugs for managing antibiotic resistance. Medicinal plants are primarily used in traditional medical systems, which for thousands of years have played an important role in resisting various diseases and caring for human health [15]. Some antibacterial components isolated from medicinal plants could have a synergistic effect with antibiotics used in hospitals; in particular, they are a new alternative to the treatment of infectious diseases caused by antibiotic-resistant bacteria [16].

Korean ginseng (*Panax ginseng* C. A. Meyer) is a valuable medicinal plant belonging to the *Panax* genus of Araliaceae, and it is well-known for a variety of ginsenosides that show diverse biological activities. Recently, many studies reported ginseng to have multiple medicinal and pharmacological properties, including an influence on the endocrine, immune, and nervous systems, and anticancer, anti-inflammation, and antiaging effects [17,18]. The components of ginsenosides are quite different depending on the processing method of Korean ginseng roots, such as dried white ginseng without processing, and processed red ginseng and black ginseng (BG) by special methods such as steam treatment [17,19]. Korean ginseng contains dammarane-type ginsenosides and a unique saponin found in the *Panax* genus that is nontoxic and displays antibacterial activity against nonhemolytic bacteria [20]. Ginsenosides Rb1, Rb2, Rc, Rd, Rg1, and Re, which are major constituents of ginseng, are transformed into less polar ginsenosides Rg3, Rg5, Rk1, and F4 according to the processing method [21]. BG is usually developed from fresh or white ginseng steamed several (usually 9) times at 96 °C for 3 h, followed by hot-air-drying at 50 °C for 24 h [22,23,24,25]. While this processing method has always been used in Korea in the processing of Rehmanniae Radix (*Rehmannia glutinosa* Liboschitz ex Steudel; Scrophulariaceae), it has been rarely used for processing ginseng in ancient Eastern medicine [26,27]. Black ginseng was first prepared in South Korea; then, it became widely used in China and Southeast Asian countries [26]. Previous studies reported that BG exhibits antibacterial, anticarcinogenic, immunomodulatory, anti-inflammatory, hepatoprotective, antidiabetic, antiobesity, antihyperlipidemic, antioxidant, and tonic effects [17,20,26], however, the inhibitory effect of BG on MRSA toxins is not yet clear. In this study, antivirulence activity was compared according to BG processing method (steamed and dried 3 or 5 times, respectively).

## 2. Results

### 2.1. Metabolite Profiling of Black Ginseng Products by UPLC-QTOF/MS

**Figure 1:**
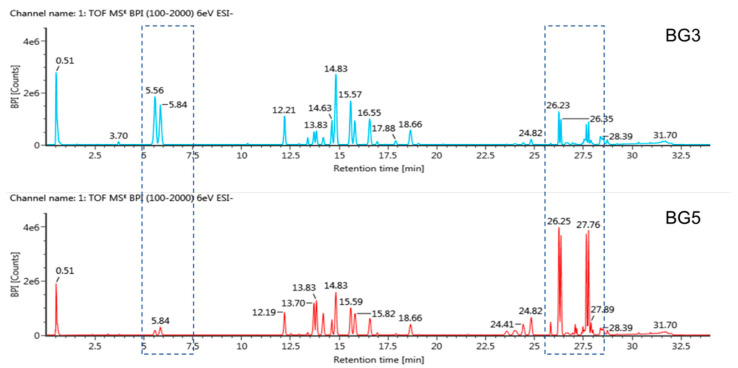
The base peak intensity (BPI) chromatogram of diverse metabolites from BG3 and BG5.

With an extraction method constructed in our previous study [28], UPLC-QTOF/MS was used to profile various metabolites, including ginsenosides, from BG3 and BG5. Methanol (70% *v*/*v*) was used to extract metabolites from the samples, and then, the extracts were subjected to UPLC-QTOF/MS in the negative ion mode. Figure 1 shows the representative base peak intensity (BPI) chromatograms of diverse metabolites from BG3 and BG5, respectively. The intensity of several peaks was different depending on distinct samples. For example, compared to other products, BG5 showed a lower intensity of peaks eluted from 5.56 to 5.84 min and higher intensity of peaks eluted from 26.25 to 27.76 min in the BPI chromatogram. These demonstrated that different processing methods changed the metabolic composition of BG. Moreover, this information is necessary for the reproducibility of the work.

### 2.2. Growth Curve Analysis for Methicillin-Resistant Staphylococcus aureus

**Figure 2:**
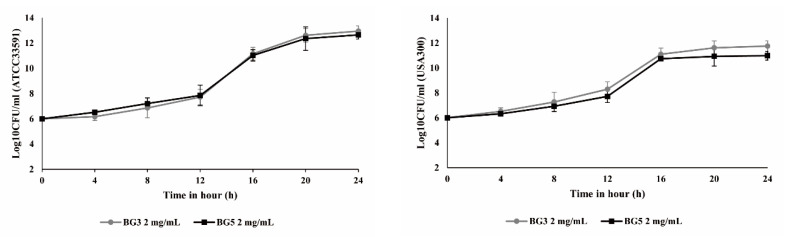
Growth curve for methicillin-resistant *Staphylococcus aureus* (MRSA) cultured with subinhibitory concentrations of BG3 or BG5. Bacterial suspensions (final density, 1.5 × 10^6^ CFU/mL) were incubated with 2 mg/mL of BG3 or BG5. At about 4 h intervals, aliquots (0.1 mL) of the culture were serially diluted 10-fold in saline as needed and plated on TS agar, followed by incubation at 37 °C for 24 h. Colony counts were performed on plates, and 30–300 colonies were enumerated. Each point of each group represents the means ± standard deviation of three independent experiments, and the variance of each data did not exceed 5% relative to the mean value.

The ATCC 33591 was a hospital-associated MRSA (HA-MRSA) standard strain, and USA300 was a wild-type LA County (LAC) strain, community-associated MRSA (CA-MRSA) [29]. The USA300 used in this study is resistant to methicillin, and was obtained from the Advanced Radiation Technology Institute, Korea Atomic Energy Research Institute (Jeongeup, South Korea) [30]. The result of the broth minimum inhibitory concentration (MIC) experiment was confirmed to be 4 mg/mL, regardless of the number of steam and drying processes (data not shown). Growth curve analysis treated for 0, 4, 8, 12, 16, 20, and 24 h with 1/2 MIC (2 mg/mL) of BG extract also showed no growth inhibition, respectively (Figure 2). These results show that BG does not have high antibacterial activity against MRSA. However, we hypothesized that BG may reduce the ability of *S. aureus* toxins to attack the host; therefore, to investigate this, we tested the effect of BG extract on toxicity-related factors in MRSA.

### 2.3. Inhibition of TNF-α Expression of BG Extracts in RAW 264.7 Cells

**Figure 3:**
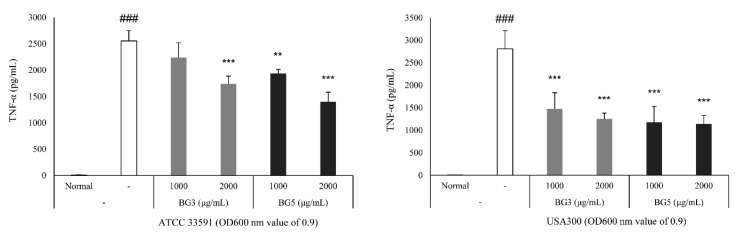
Effect of BG3 and BG5 in TNF-α secretion of 264.7 RAW macrophages stimulated by methicillin-resistant *Staphylococcus aureus* (MRSA)-cultured media. After stimulation for 16 h with supernatants of MRSA grown in the presence of graded concentrations of BG3 or BG5 in RAW 264.7 cells, TNF-α levels were measured by enzyme-linked immunosorbent assay (ELISA). Normal group refers to RAW 264.7 cells not treated MRSA-cultured media or BG extracts. Values represent the mean and standard error of three independent experiments. ^###^ *p* < 0.001 in comparison with normal group. ^**^ *p* < 0.01 and ^***^ *p* < 0.001 in comparison with the MRSA group.

To clarify the biological relevance of the reduction of Staphylococcal exotoxin secretion by BG, we performed a tumor necrosis factor (TNF) release assay. The expression level of TNF-α was measured in RAW 264.7 cells stimulated by MRSA-cultured media [31,32]. Among the virulence factors secreted by *S. aureus*, α-toxin is primarily responsible for the hemolytic activity, and enterotoxins are the most important exotoxins that could act as superantigens, stimulating T cells to release proinflammatory cytokines [32,33]. Graded concentrations of BG (1000 and 2000 μg/mL) were added to the diluted bacterial suspensions. MRSA supernatant without BG treatment was used as a control. After 4 h incubation, the supernatants (700 μL) collected (protein secretion) were filtered through a 0.2 μm filter and immediately analyzed. As a result, the expression of TNF-α decreased in cells stimulated by an MRSA culture medium treated with BG extract, and the BG5 extract inhibited more than the BG3 extract did (Figure 3).

### 2.4. Inhibitory Effect of BG Extracts in Toxin Expression of S. aureus

**Figure 4:**
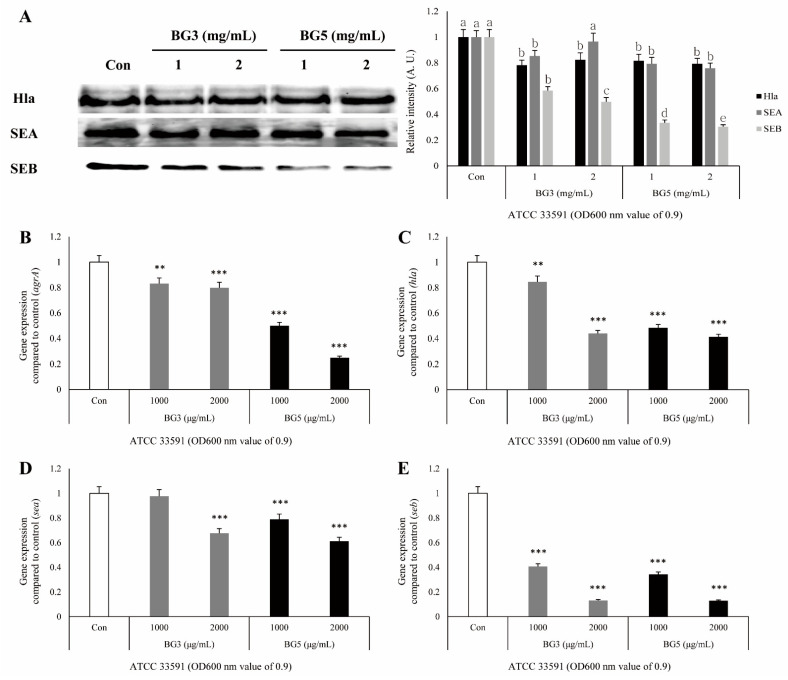
(**A**) Inhibitory effects of BG3 and BG5 on the expression of toxic proteins including α-hemolysin (Hla), staphylococcal enterotoxin A (SEA), and staphylococcal enterotoxin B (SEB) in *S.* *aureus* ATCC33591, as analyzed by Western blotting. *S. aureus* ATCC 33591 cells treated with serial dilutions of BG3 or BG5 (1 and 2 mg/mL) for 4 h. (a–e) Significant differences at *p* < 0.05 level according to the analysis of variance followed by Scheffe’s test for multiple comparisons (*p* < 0.05). Values presented as mean ± SD. (**B**–**E**) The inhibitory effect of BG3 and BG5 on the expression of mRNA of *hla*, *agrA*, *sea*, and *seb* as analyzed by qRT–PCR. *S. aureus* ATCC 33591 treated with serial dilutions of BG 3 or 5 for 4 h. Values represent the mean and standard error of three independent experiments. ^**^ *p* < 0.01 and ^***^ *p* < 0.001 in comparison with MRSA (control) group.

**Figure 5:**
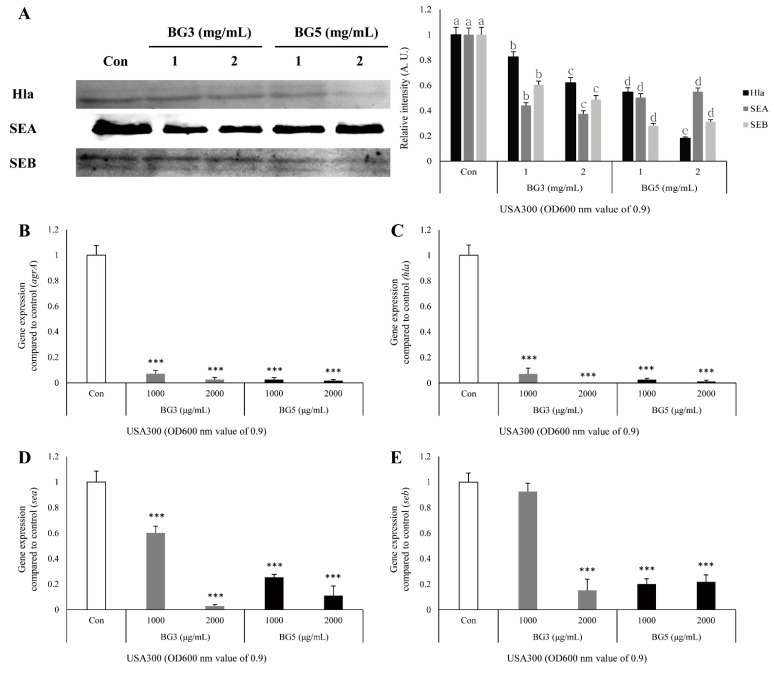
(**A**) Inhibitory effects of BG3 and BG5 on α-hemolysin (Hla), staphylococcal enterotoxin A (SEA), and staphylococcal enterotoxin B (SEB) production in *S. aureus* USA300, as analyzed by Western blotting. *S. aureus* USA300 cells treated with serial dilutions of BG3 or BG5 (1 and 2 mg/mL) for 4 h. (a–e) Significant differences at *p* < 0.05 level according to the analysis of variance followed by Scheffe’s test for multiple comparisons (*p* < 0.05). Values, means ± standard errors. (**B**–**E**) The inhibitory effect of BG3 and BG5 on the expression of mRNA of *hla*, *agrA*, *sea*, and *seb* as analyzed by qRT–PCR. *S. aureus* USA300 treated with serial dilutions of BG 3 or 5 for 4 h. Values represent mean and standard error of three independent experiments. ^***^ *p* < 0.001 in comparison with MRSA (control) group.

Western blotting was performed to investigate the effect of BG against toxin expression in protein levels. Results showed that MRSA toxins were inhibited by treating with BG. In *S.* *aureus* ATCC33591, Hla and SEA were slightly inhibited by treating with BG. However, SEB was significantly inhibited by treating with BG, and BG5 was more effective than BG3 was (Figure 4A). In *S. aureus* USA300, three toxins were significantly inhibited by treating BG. Hla and SEB were especially decreased more by treating BG5 than by treating BG3. In contrast, SEA was inhibited more by treating BG3 than by treating BG5 (Figure 5A).

### 2.5. Inhibitory Effect of BG Extracts on Expression of mRNA-Associated Virulence Factor of MRSA

It was confirmed by quantitative real-time polymerase chain reaction (qRT-PCR) that antivirulence activities, including on the *hla* and *agrA* genes related to Hla in MRSA culture medium, were associated with mRNA reduction by BG extract. The expression of the four genes (*hla, sea, seb,* and *agrA*) was significantly suppressed in *S. aureus* ATCC 33591 and USA300 by the treatment of BG extracts. The 16S ribosome RNA (rRNA) was used as a reference gene. In *S. aureus* ATCC 33591, it was confirmed that the inhibitory effect of BG5 extract was greater than that of BG3 extract against virulence-related gene expressions (Figure 4B–E). In *S. aureus* USA300, both BG3 and BG5 showed high inhibitory effects on the *hla* and *agrA* genes. In the expression of *sea* and *seb* genes, the concentration of 2 mg/mL of BG3 extract showed the best inhibitory effect (Figure 5B–E).

## 3. Discussion

Ginseng has been widely used in Eastern medicine for thousands of years, and white ginseng, prepared by drying fresh ginseng, is steamed again and dried to produce black and red ginseng. During this process, the ingredients of fresh ginseng are changed, and new physiological ingredients (20(S)-, 20(R)-Rg3, Rk3, Rh4, Rk1, Rg5, etc.) that are not present in fresh ginseng are generated and their content is increased [24]. Generally, white ginseng is manufactured by the dehydration of fresh ginseng using sunlight, and red ginseng is produced by steaming fresh ginseng at 95–100 °C for a reasonable amount of time [23]. The processing method of BG still requires in-depth research, but it takes at least three more steaming and drying processes than red ginseng does. In detail, when processing *Radix Rehmanniae* in traditional Eastern medicine, the process of steaming and drying is repeated nine times, and this process is modified and used for ginseng. In Korea, this processing method is called kujeung-kupo, meaning that it is steamed nine times and dried nine times. However, a standard process for the production of black ginseng has not yet been established, and this kujeung-kupo process is occasionally found to produce excessive benzopyrene, which was recently classified as a carcinogen [24]. Therefore, the aim of this study was to explore new functionalities that can suppress the toxicity of MRSA while being safe using BG made by a temporary process that repeats the steaming and drying process 3 or 5 times.

MRSA is the most common cause of hospital-associated multidrug-resistant infections, and there is a very high risk of infection in people with impaired immunity, such as weakened immunity, an underlying disease, or a disrupted skin barrier [34]. Moreover, today’s MRSA is no longer limited to inpatients or persons with predisposing risk factors, and it is globally reported in diverse community populations [29,35]. CA-MRSA strains are recognized as distinct clonal entities that differ from traditional HA-MRSA strains, and both CA-MRSA and HA-MRSA are resistant to methicillin (and all beta-lactam antibiotics), but with important differences, such as in epidemiology, microbiologic characteristics, clinical aspects of infection, and management [35]. Therefore, it is necessary to understand the antibiotic resistance and virulence factors of bacteria and to develop antibacterial agents with new mechanisms.

Hla, also known as α-toxin, is the major cytotoxic agent released by the *S. aureus* bacterium and the first identified member of the pore-forming beta-barrel toxin family [36]. *S. aureus* exoproteins, such as exotoxins and enzymes, convert the host tissue into nutrients for bacterial growth and possess properties including pyrogenicity and superantigenicity [32]. Hla significantly contributes to *S. aureus*-induced cell death and triggers caspase activation via the intrinsic death pathway, independently of death receptor signaling [32,37]. SEs, as superantigens, can result in staphylococcal gastroenteritis, one of the causes of food poisoning in humans [32,38]. SEs secreted by *S. aureus* stimulate cells in the immune system, such as macrophages, to release TNF and other proinflammatory cytokines [39]. Therefore, a TNF-α release assay was carried out to elucidate the biological relevance of the reduction in Hla, SEA, and SEB secretion induced by BG extracts. The secretion of TNF-α in RAW264.7 cells treated with MRSA-culture media was increased, and this response was attenuated by treatment with BG extracts (Figure 3). These results suggest that BG extracts inhibit the activation of immune responses, which are activated by the virulence factors derived from MRSA. It is known that the expression of the *hla* gene is regulated by the *agrA* gene. Agr quorum sensing is an important bacterial regulatory system by detecting extracellular autoinducers, and is responsible for regulating the virulence of many bacterial pathogens [14]. It is also associated with an increased expression of virulence genes encoding toxins and degradative exoenzymes, which are essential for the establishment of infection [40,41,42,43]. Therefore, we examined the alteration of protein expression of Hla, SEA, and SEB by Western blot analysis, and mRNA expression of *agrA*, *hla*, *sea,* and *seb* by quantitative reverse-transcription polymerase chain reaction (qRT-PCR), which are important factors in the pathogenicity of MRSA. Treatment with BG extract resulted in the decreased protein expression of Hla, SEA, and SEB in MRSA. In addition, BG extract was shown to decrease *agrA*, *hla*, *sea*, and *seb* expression in MRSA (Figure 4 and Figure 5).

Therefore, BG strongly reduces the secretion of selected virulence factors produced by *S. aureus*, suggesting that a BG-based structure may be used for the development of drugs aimed at staphylococcal virulence-related exoproteins.

## 4. Materials and Methods

### 4.1. BG Extract Preparation

In this study, 5-year-old fresh Korean ginseng roots (Chungcheongbuk-do, Republic of Korea) available at the market were used as a sample. The optimal conditions for the preparation of BG were manufactured by 3 (BG3) and 5 (BG5) time repeated steaming of the white ginseng at 95–98 °C for 5 h in pottery apparatus, followed by drying at 50 °C for 24 h. The dried BG was pulverized to be fine enough to pass through mesh 80. BG was coarsely ground and extracted twice by reflux extraction with 30% fermented ethanol at 80 °C for 4 h. After extraction, extracts were filtered using a filter paper (No. 1, Whatman, Maidstone, UK) and using a decompression distillation apparatus (N-1000, EYELA, Tokyo, Japan). The concentrated extract was obtained using a −80 °C ultralow-temperature freezer (DF8520, IlshinBioBase, Dongducheon, Korea) and freeze dryer (FDU-1200, EYELA, Tokyo, Japan). 

### 4.2. Bacterial Strains and Reagents

The *S. aureus* ATCC 33591 strain used in this study was a standard strain of HA-MRSA, and the *S. aureus* USA300 strain is now the dominant CA-MRSA strain in several countries, a major international epidemic clone that causes both community- and hospital-onset infections [44]. *S. aureus* ATCC 33591 was incubated in brain–heart infusion (BHI), and *S. aureus* USA300 was incubated in tryptic soy (TS) broth at 37 °C for 24 h. RAW 264.7 (macrophage, ATCC TIB-71) cells were cultured in an RPMI 1640 medium supplemented with 10% fetal bovine serum and 100 U/mL of penicillin/streptomycin sulfate. Cells were cultured in a humidified incubator with 5% CO_2_ atmosphere at 37 °C. Primary antibodies, including anti-*Staphylococcus* alpha hemolysin antibody, rabbit polyclonal anti-*Staphylococcus* enterotoxin A antibody, and rabbit polyclonal anti-*Staphylococcus* enterotoxin B antibody were purchased from Abcam (Cambridge, UK). In addition, anti-rabbit IgG and goat anti-mouse IgG secondary antibodies were obtained from Thermo Scientific (Wilmington, DE, USA).

### 4.3. Measurement of Minimum Inhibitory Concentration, and Bacterial Growth Curve Assay

Serial twofold dilutions of BG extracts were prepared in 100 µL of BHI and TS broth in 96-well microplates. Microplates were inoculated with MRSA adjusted to a 0.5 McFarland standard (approximately 10 µL of 1.5 × 10^8^ colony-forming units (CFU)/mL; final density, 1.5 × 10^6^ CFU/mL). The MIC was determined as the lowest BG concentration suppressing the growth of MRSA after 24 h of incubation at 37 °C. Bacterial cells were harvested at an early log phase (OD_600_ = 0.5) and resuspended in phosphate-buffered saline (PBS) at ~10^8^ to 10^9^ CFU/mL. Subsequently, bacterial samples were incubated with 1/2 MIC of BG at different intervals up to 24 h at 37 °C. After incubation, bacterial samples were applied on the surface of TS agar plates. After 16 h of incubation at 37 °C, the surviving bacterial cells were counted.

### 4.4. Western Blot Analysis

The proteins of Hla, SEA, and SEB were detected by a Western blot analysis. The detailed procedures are carried out based on the previously reported investigation [27]. Briefly, MRSA strain *S. aureus* ATCC 33591 or USA300 was grown to an OD_600_ value of 0.9 in BHI or TS broth with various concentrations of BG, and the bacterial cells were prepared with SMART^TM^ bacterial protein-extraction solution (iNtRON Biotechnology) according to the manufacturer’s protocol. 20 μL of protein lysates were separated by 12% sodium dodecyl sulfate polyacrylamide gel electrophoresis (SDS-PAGE), and the resolved proteins were transferred onto polyvinylidene difluoride membranes. The membrane was blocked with 5% skimmed milk and sequentially incubated with the primary antibodies (1:500) and secondary antibodies (1:2500). Immunoreactive protein bands were detected using the EzWestLumi Plus luminol substrate (ATTO Co., Tokyo, Japan), and visualized using an ImageQuant LAS 4000 mini chemical luminescent imager (GE Healthcare Korea, Seoul, Korea).

### 4.5. Tumor Necrosis Factor-α Enzyme-Linked Immunosorbent Assay (ELISA)

The detailed procedures for this assay are carried out based on the previously reported investigation [27]. Briefly, the MRSA was grown to an OD_600_ value of 0.9 in Mueller-Hinton (MH) broth with various concentrations of BG extract. After 4 h incubation, the supernatants were collected by filtering through a 0.2 μm filter. RAW 264.7 cells were seeded at a density of 10^6^/mL in RPMI, dispensed (100 μL) into 96 well tissue culture plates, and incubated in 5% CO_2_ at 37 °C in an incubator for 18 h to allow for adherence. After cell culture media were washed, the RPMI 1640 medium (150 μL) and the supernatants of the strain (*S. aureus* ATCC 33591 and USA300, 50 μL) were added to the tissue culture plate. After incubation for 16 h, the culture media were collected to determine the levels of TNF-α present in each sample using the ELISA kit from OptEIA^TM^ human enzyme-linked immunosorbent assay (ELISA; BD Bioscience, San Jose, CA, USA) according to the manufacturer’s instruction. 

### 4.6. RNA Extraction and Quantitative Reverse-Transcription–Polymerase Chain Reaction (qRT-PCR)

Total RNA was isolated from *S. aureus* ATCC 33591 or USA300 using an E.Z.N.A.^®^ bacterial RNA kit (Omega Bio-tek, Norcross, GA, USA) according to the manufacturer’s instructions. Total RNA was dissolved in diethyl pyrocarbonate-treated distilled water. The concentration of RNA was determined by measuring the absorbance ratio at 260 and 280 nm on a spectrophotometer (BioTek, Winooski, VT, USA). Complementary DNA was synthesized using the QuantiTect reverse transcription kit (Qiagen, Seoul, Korea) according to the manufacturer’s instructions. The sequences of the primers used in this experiment are listed in Table 1. The reaction was performed in triplicate using a Power SYBR^®^ Green PCR master mix (Applied Biosystems, Foster City, CA, USA) and a StepOnePlus real-time PCR system (Applied Biosystems). The data were analyzed and the expression levels of target genes relative to the endogenous reference gene, 16S rRNA, were calculated by the delta–delta cycle threshold method, and the data were analyzed using StepOne software (Applied Biosystems).

### 4.7. Statistical Analysis

All results are presented as mean ± standard error of three independent experiments. Statistical analysis was performed with GraphPad Prism software, version 4 (GraphPad Software Inc., San Diego, CA, USA). Tukey’s multiple-comparison test was applied for statistically significant differences between mean values (*p* < 0.05). The TNF-α levels of statistical analysis were established by Scheffe’s test (SPSS. ver.23) for multiple comparisons. *p* values < 0.05 were considered significant. 

## 5. Conclusions

In the present study, we analyzed changes in antivirulence activities depending on the number of rounds of steaming and drying for Korean ginseng. BG extracts showed weak antibacterial activity with high MIC values against MRSA strains. However, the treatment with BG extracts inhibited the protein and mRNA expression of virulent factors including Hla, SEA, SEB, and agrA against MRSA strains. These results suggest that it may be a candidate for treatment or a combination of MRSA infection. We next plan to study the mechanism of this activity and various physiological activities of BG.

## Figures and Tables

**Table 1:** Primer sequences for real-time RT-PCR.

Target Genes	Primer Sequences (5′–3′)	
	Forward Primer	Reverse Primer
*hla*	TTGGTGCAAATGTTTC	TCACTTTCCAGCCTACT
*sea*	ATGGTGCTTATTATGGTTATC	ATGGTGCTTATTATGGTTATC
*seb*	TGTTCGGGTATTTGAAGATGG	CGTTTCATAAGGCGAGTTGTT
*agrA*	TGATAATCCTTATGAGGTGC	CACTGTGACTCGTAACGAAA
16s rRNA	CGTGCTACAATGGACAATAC	ATCTACGATTACTAGCGATT

## Data Availability

No new data were created or analyzed in this study. Data sharing is not applicable to this article.
